# A PET Radiomics Model to Predict Refractory Mediastinal Hodgkin Lymphoma

**DOI:** 10.1038/s41598-018-37197-z

**Published:** 2019-02-04

**Authors:** Sarah A. Milgrom, Hesham Elhalawani, Joonsang Lee, Qianghu Wang, Abdallah S. R. Mohamed, Bouthaina S. Dabaja, Chelsea C. Pinnix, Jillian R. Gunther, Laurence Court, Arvind Rao, Clifton D. Fuller, Mani Akhtari, Michalis Aristophanous, Osama Mawlawi, Hubert H. Chuang, Erik P. Sulman, Hun J. Lee, Frederick B. Hagemeister, Yasuhiro Oki, Michelle Fanale, Grace L. Smith

**Affiliations:** 1 Department of Radiation Oncology, Division of Radiation Oncology, MD Anderson Cancer Center, Houston, TX USA; 2 Department of Radiation Physics, Division of Radiation Oncology, MD Anderson Cancer Center, Houston, TX USA; 3 Department of Genomic Medicine, The University of Texas MD Anderson Cancer Center, Houston, TX USA; 4 Department of Translational Molecular Pathology, Division of Pathology/Lab Medicine, The University of Texas MD Anderson Cancer Center, Houston, TX USA; 5 Department of Bioinformatics and Computational Biology, Division of Quantitative Sciences, The University of Texas MD Anderson Cancer Center, Houston, TX USA; 6 Department of Imaging Physics, Division of Diagnostic Imaging, The University of Texas MD Anderson Cancer Center, Houston, TX USA; 7 Department of Nuclear Medicine, Division of Diagnostic Imaging, The University of Texas MD Anderson Cancer Center, Houston, TX USA; 8 Department of Lymphoma/Myeloma, Division of Cancer Medicine, The University of Texas MD Anderson Cancer Center, Houston, TX USA

## Abstract

First-order radiomic features, such as metabolic tumor volume (MTV) and total lesion glycolysis (TLG), are associated with disease progression in early-stage classical Hodgkin lymphoma (HL). We hypothesized that a model incorporating first- and second-order radiomic features would more accurately predict outcome than MTV or TLG alone. We assessed whether radiomic features extracted from baseline PET scans predicted relapsed or refractory disease status in a cohort of 251 patients with stage I-II HL who were managed at a tertiary cancer center. Models were developed and tested using a machine-learning algorithm. Features extracted from mediastinal sites were highly predictive of primary refractory disease. A model incorporating 5 of the most predictive features had an area under the curve (AUC) of 95.2% and total error rate of 1.8%. By comparison, the AUC was 78% for both MTV and TLG and was 65% for maximum standardize uptake value (SUV_max_). Furthermore, among the patients with refractory mediastinal disease, our model distinguished those who were successfully salvaged from those who ultimately died of HL. We conclude that our PET radiomic model may improve upfront stratification of early-stage HL patients with mediastinal disease and thus contribute to risk-adapted, individualized management.

## Introduction

In early-stage classical Hodgkin lymphoma (HL), individualized, risk-adapted therapy is desirable to maintain high cure rates while minimizing treatment-related toxicity. For most patients, prognosis is excellent, so de-intensification of therapy is advised to reduce the risk of late treatment-related toxicity^1,2^. However, a small minority of patients develop relapsed or refractory disease, which may be fatal. Primary refractory disease is associated with particularly poor outcomes^3,4^. For patients with refractory disease, therapy should not be minimized^5^. Thus, upfront identification of patients at high risk for refractory disease would be extremely valuable.

The intensity of frontline therapy is dictated by the presence of risk factors at the time of diagnosis. In today’s practice, these risk factors are defined using historic methods. In particular, tumor bulk has long been recognized as an important poor prognostic factor^6^. Historically, bulk was defined based on the width of the mediastinum on an upright chest x-ray^7,8^. Subsequently, in the era of computed tomography (CT) scanning, various retrospective studies defined CT criteria for bulky disease, with proposed cut-off values ranging from 5 to 10 cm^9–12^. Most recently, measurements that reflect both the 3-dimensional disease volume and metabolic activity, such as metabolic tumor volume (MTV) and total lesion glycolysis (TLG), have been associated with patient outcomes in HL^13–15^. These functional measurements of tumor volume provide additional prognostic information beyond the classical risk factors that include a unidimensional measurement of tumor bulk^13^. While these metabolic tumor volume metrics may improve risk stratification, further study and validation are needed before they can be incorporated into current daily practice and risk stratification systems.

As a next step, additional data beyond MTV and TLG may be derived from ^18^F-fluorodeoxyglucose (FDG) positron emission tomography (PET) scans. Radiomics involves the extraction of a large number of quantitative features from digital images, which can be mined for hypothesis generation^16,17^. When extracted from PET scans, these features signify aspects of radiotracer intensity (concentration), heterogeneity, and shape within the tumor, reflecting biological characteristics, such as cellular density, proliferation rate, hypoxia, necrosis, and angiogenesis^18^. “First-order” features are global measurements that do not convey spatial information, such as standardized uptake value (SUV), MTV, and TLG. Second-order features reflect spatial relationships between ≥2 voxels due to variability in the distribution of radiotracer uptake. These data can be derived from gray-level co-occurrence matrices (GLCM). Examples of second-order features include contrast, energy, entropy, and homogeneity^19^. In multiple other malignancies, first- and second-order features of baseline PET scans have been associated with patient outcomes^20–31^. Furthermore, textural and shape parameters evaluated on baseline PET scans predicted early metabolic response in a cohort of patients with bulky Hodgkin and non-Hodgkin lymphoma^32^. We hypothesized that a model incorporating first- and second-order radiomic features would more accurately predict refractory or relapsed disease status, when compared to MTV, TLG, or maximum SUV (SUV_max_) alone, in a cohort of early-stage HL patients.

## Materials and Methods

We obtained approval from the University of Texas MD Anderson Cancer Center Institutional Review Board. All work was done in accordance with institutional guidelines and regulations. The status of this study as a HIPAA-compliant, retrospective project waived the prerequisite for informed consent.

### Study cohort

Records were reviewed retrospectively for all patients treated at a single tertiary cancer center for classical HL from 2003 through 2013. Inclusion criteria included Ann Arbor stage I or II disease, age of ≥18 years at the time of diagnosis, and availability of an analyzable pre-chemotherapy PET-CT scan. Patients with a disease volume measuring <5 cc were excluded.

### PET-CT scans

PET-CT scans were acquired on 1 of 4 scanners: a DST machine, 2 DRX machines, or a DSTE machine (GE Healthcare, Milwaukee, WI). The corresponding CT scanners were 8-slice (DST model), 16-slice or 64-slice (DRX model), or 64-slice machines (DSTE model). All PET-CT scanners used the same DISCOVERY platform by General Electric.

Patients fasted for at least 6 hours and were confirmed to have a blood glucose level of <150 mg/dL prior to the FDG injection. An intravenous FDG injection of 555–629 MBq (15–17 mCi) or of 333–407 MBq (9–11 mCi) was administered for 2-dimensional (2D) and 3-dimensional (3D) imaging, respectively, and emission scans were acquired at 3 minutes per field of view. The injection-to-scan time of all patients had a median of 70 minutes (standard deviation 17 minutes). PET images were reconstructed with vendor-provided algorithms. Diagnostic quality CT images were acquired in helical mode with a 3.75-mm slice thickness (pitch of 1.35, rotation speed of 0.5 sec, kVp of 120, and noise index of 30).

All PET-CT scanners at our institution are subject to a rigorous quality assurance/quality control program on a daily, quarterly, and annual basis. Additionally, reconstruction parameters are optimized to ensure harmonization of SUV measurements between scanners. PET data were acquired in 2D mode before January 2008 and in 3D mode after that date. The equivalence between the 2D and 3D reconstruction data have been confirmed using phantom data.

### Disease volume definition

PET-CT images were transferred to MIM software (version 6.4.9, MIM Software Inc., Cleveland, OH) and co-registered for further analysis. All sites of nodal disease were contoured manually on the CT scan. Disease was contoured separately by anatomic region, as defined by the Ann Arbor staging method (i.e. mediastinum, left neck, right neck, left axilla, right axilla, etc). Then, an auto-thresholding technique was used to delineate all tumor on the PET scans that was present within the manually contoured volumes and had a body weight SUV ≥2.5^33^.

MTV and TLG were defined using the fixed SUV threshold of 2.5, with all voxels containing SUVs above this cut-off contributing to the MTV. MTV_2.5_ was calculated in cubic centimeters (cc) by summation of these voxels. For cases in which SUV_max_ of the primary lesion was lower than that of the threshold, MTV_2.5_ was considered to be 0. TLG_2.5_ was computed by multiplying the MTV_2.5_ by its SUV_mean_.

### Radiomics analysis

Thirty-three quantitative radiomic features, listed in Table 1, were extracted from the MTV_2.5_ using the in-house imaging software “Imaging Biomarker Explorer” (IBEX)^34^. This software was designed based on the MATLAB (v8.1.0, MathWorks, Natick, MA) and is available at http://bit.ly/IBEX.MDAnderson. Any disease volume <5 cc was excluded, because quantitative features extracted from small lesions yield less reproducible results than those from larger tumors^35^. Clean MaskEdge was applied before feature extraction to specify how much of the edge voxel to include in the calculations. IBEX can extract features from discontinuous regions of interest, so all involved nodes within the site of interest were included. Texture features were derived from the GLCM in 2.5D (i.e. first computed on each 2D image and then combined into 2.5D features by averaging of 2D features across the 3D volume).

**Table 1:** Radiomic features.

Category	Features			
**Intensity histogram**	Global Entropy Global Uniformity	Global Max Inter quartile Range	Global Mean Kurtosis	Global standard deviation Skewness
GLCM2.5D*	Auto Correlation Contrast Energy Information Measure 1 Inverse Variance Sum Variance	Cluster Prominence Correlation Entropy Information Measure 2 Max probability Variance	Cluster Shade Difference Entropy Homogeneity Inverse Diff. Norm Sum Average	Cluster Tendency Dissimilarity Homogeneity 2 Inverse Diff. Moment Norm Sum Entropy
**Shape**	Max 3D Diameter	Volume	Roundness	

*Gray-Level Co-Occurrence Matrix, computed in 2.5D fashion.

### Clinical outcomes

The primary clinical outcome was refractory or relapsed disease, with the clinical rationale that patients who suffer refractory or relapsed disease outcomes are at higher risk for mortality, need for salvage chemotherapy, and need for stem cell transplantation^3,4,36,37^. Primary refractory disease was defined as persistent HL during or within 90 days of completion of upfront therapy (i.e. the final cycle of chemotherapy in patients treated with chemotherapy alone, or the final day of RT in patients treated with combined modality therapy). Relapsed disease was defined as progression more than 3 months after upfront therapy. As a secondary outcome, we assessed death from HL.

### Statistical analysis

We aimed to build an imaging biomarker-based model predictive of relapsed or refractory HL. We hypothesized that this model would more accurately predict patient outcomes than MTV, TLG, or SUV_max_. Training-validation subsets were used to identify prognostic radiomic features. To investigate the association of primary refractory disease with mediastinal radiomic features specifically, patients with mediastinal involvement (167 patients, 12 refractory cases) were separated randomly into 12 groups, with 1 refractory case per group. Ten training groups established a support vector machine-based AdaBoost iterating algorithm enhanced classifier^38^, and the remaining 2 groups tested the model.

Receiver operating characteristic (ROC) curves were used to test the predictive performance of this model, compared to single variable MTV-, TLG-, and SUV_max_-based prediction. Classifier and statistical analyses were built and performed using R3.2.2. The clustering analysis was conducted based on the unsupervised hierarchical clustering via pheatmap R package with default parameters.

## Results

The cohort comprised 251 patients with stage I-II HL. Patient characteristics are summarized in Table 2. The radiomic features listed in Table 1 were extracted from the baseline PET scans. We assessed for an association of these features with the presence of relapsed or refractory disease.

**Table 2:** Patient, treatment, and disease characteristics.

Characteristic	Total Cohort (n = 251)	Subset with Mediastinal Disease (n = 169)	Subset with Refractory Mediastinal Disease (n = 12)
Median age (range)	31 years (18–88)	30 years (18–60)	29 years (20–57)
Female	144 (57%)	113 (67%)	7 (58%)
Median Karnofsky performance status at diagnosis (range)	90% (70–100%)	90% (70–100%)	90% (80–100%)
Stage I/Stage II	37 (15%)/214 (85%)	8 (5%)/161 (95%)	0 (0%)/12 (100%)
B symptoms	57 (23%)	40 (24%)	4 (33%)
Bulk (>10 cm)	76 (30%)	55 (33%)	7 (58%)
Extranodal disease	11 (4%)	6 (4%)	1 (8%)
ABVD or ABVD-like chemotherapy	246 (98%)	166 (98%)	11 (92%)
Median number of chemotherapy cycles (range)	5 (2–6)	6 (2–6)	6 (2–6)
Consolidative radiation therapy as part of frontline therapy	175 (70%)	116 (69%)	1 (8%)
Primary refractory cases	19 (8%)	12 (7%)	12 (100%)
Relapsed cases	9 (4%)	7 (4%)	

ABVD = doxorubicin, bleomycin, vinblastine, dacarbazine.

**Figure 1:**
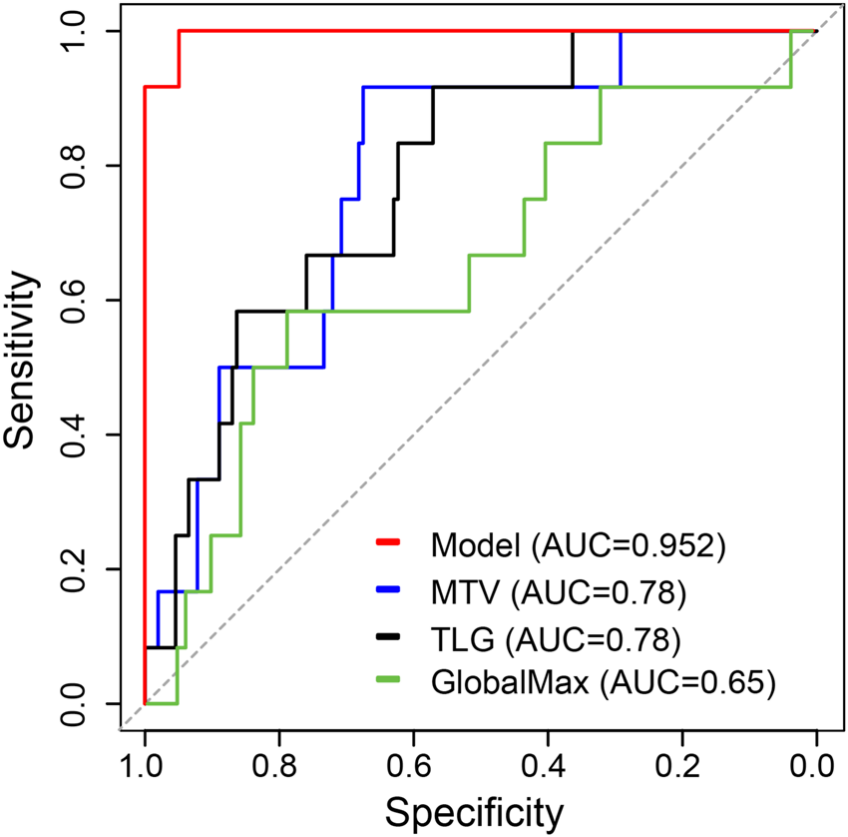
Receiver Operating Curves for the model incorporating 5 radiomic features (red), metabolic tumor volume (blue), total lesion glycolysis (black), and GlobalMax (SUVmax, green) in the subset of patients with mediastinal disease.

Radiomic features extracted from mediastinal sites on the pre-treatment scans were highly predictive of primary refractory status. Of the 169 patients with mediastinal involvement, 12 had refractory disease. In each case, the primary refractory site included the mediastinum; in 2 patients, refractory disease was present within the neck, as well. When considering mediastinal sites only (n = 169), the baseline radiomic features that were most predictive of primary refractory disease included the first-order features GlobalMax (i.e. SUV_max_) and Volume, and the second-order features InformationMeasureCorr1, InformationMeasureCorr2, and InverseVariance derived from the GLCM_2.5_. Background information regarding these features is summarized in Table 3. These 5 features were included in a model that predicted the risk of primary refractory HL with an area under the curve (AUC) of 95.2% (95% CI: 87.0–100.0%) and total error rate of 1.8%. By comparison, the AUC was 78% (95% CI: 66.6–90.3%) for both MTV and TLG and was 65% (95% CI: 48.1–83.4%) for SUVmax alone (Fig. 1).

**Table 3:** PET radiomic features that were most predictive of refractory mediastinal disease.

Feature	Definition	Equation	Reference
Intensity Global Max	The intensity maximum among all the voxels (SUV_max_).		^53^
Volume	The physical volume. For positron emission tomography, volume is equivalent to the metabolically active tumor volume (MTV).		^54^
Inverse variance	Random variables are aggregated to minimize the variance of the weighted average where each random variable is weighted in inverse proportion to its variance.	${F}_{cm.inv.var}=2\sum _{i=1}^{{N}_{g}}\sum _{j > 1}^{{N}_{g}}\frac{{p}_{ij}}{{(i-j)}^{2}}$	^42^
InformationMeasureCorr1*	First measure of information theoretic correlation, where HXY is the entropy for joint probability.	${F}_{cm.info.corr.1}=\frac{HXY-\,HX{Y}_{1}}{HX}$	^55^
InformationMeasureCorr2*	Second measure of information theoretic correlation, a grey level co-occurrence textural feature.	${F}_{cm.info.corr.2}=\sqrt{1-\exp (-2(HX{Y}_{2}-HXY)}$	^55^

*Information theoretic correlation is a grey level co-occurrence textural feature and an index of tumor heterogeneity. It is estimated using 2 different measures that incorporate entropy chiefly in the computation process^56^.

**Figure 2:**
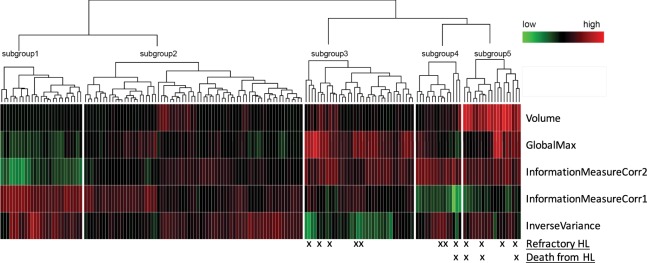
Heatmap demonstrating the prognostic subgroups based on the 5 most predictive mediastinal radiomic features.

Based on these 5 features, the expression profiles were subject to unsupervised hierarchical clustering, and the patients were divided into 5 subgroups (Fig. 2 and Table 4). No patient in Group 1 (n = 27) or 2 (n = 72) had primary refractory disease. The other 3 groups included all 12 patients with refractory disease. Furthermore, these groups distinguished patients with highly refractory disease who died of HL from those with initially refractory disease who were salvaged successfully. Group 3 (n = 36) included 5 patients (14%) with refractory disease, none of whom died of HL; Group 4 (n = 15) included 3 patients (20%) with refractory disease, 1 (33%) of whom died of HL; and Group 5 (n = 19) included 4 patients (21%) with refractory disease, 3 (75%) of whom died of HL (Table 4).

**Table 4:** Prognostic groups based on radiomic feature analysis.

Group	n	Refractory Cases (n = 12)	Deaths from Refractory HL (n = 4)
1	27	0	0
2	72	0	0
3	36	5 (14%)	0
4	15	3 (20%)	1 (33%)
5	19	4 (21%)	3 (75%)

In contrast, features extracted from the total disease volume, inclusive of all anatomic sites, did not predict the risk of refractory disease (49.5% error rate). Additionally, radiomic features did not predict the risk of relapsed disease (51% error rate for the largest volume and 49% for the highest SUV volume).

## Conclusion

We have identified a model for imaging biomarker-based risk stratification in early-stage HL patients with mediastinal disease. Our model incorporated 5 of the most highly predictive PET radiomic features. Two of these features, GlobalMax (SUV_max_) and Volume (MTV) are known prognostic markers in HL^13–15^. With the addition of 3 other features, all measures of texture, our model more accurately predicted the risk of primary refractory disease than MTV, TLG, or SUV_max_. This model not only identified the risk of primary refractory disease, but also distinguished a high-risk refractory subgroup that could not be salvaged. Thus, advanced imaging biomarkers may identify patients who would benefit from escalation vs. de-intensification of therapy. Such individualization of treatment could improve the therapeutic ratio, contributing to superior patient outcomes.

The management of early-stage HL patients is guided by the presence of known clinical and radiographic risk factors^5^. The radiographic finding included in current risk stratification schemas is tumor bulk. The definition of bulk varies among investigators and is typically defined by a single measurement of tumor diameter^8,39–41^. However, such unidimensional measurements do not fully represent disease burden. Several groups have shown that 3-dimensional assessments of disease volume, such as MTV, enable superior prediction of clinical outcomes when compared to standard risk factors, including unidimensional measurements of tumor bulk^13–15^. As the next step, our findings suggest that additional PET radiomic features may enhance the predictive capability of MTV. Consistent with our findings, Bouallègue *et al*. published a report of 57 patients with bulky lymphoma (14 HL and 43 non-Hodgkin lymphoma). In this cohort, a model incorporating baseline PET textural and shape parameters more accurately predicted disease response on interim PET scans than other factors, including MTV and histology^32^.

This work adds to a growing body of literature demonstrating an association between radiomic features and clinical outcome. Advanced radiomic features have been shown to predict treatment response in multiple other malignancies, such as sarcoma, breast cancer, non-small cell lung cancer, head-and-neck cancer, esophageal cancer, and cervical cancer^22–31,42,43^. In other malignancies, some of the optimal models have incorporated both PET and CT features^25^ or pre- and post-therapy imaging findings^28^, suggesting possible future directions for our work.

In this analysis, we found that baseline PET radiomic features predicted the risk of refractory disease, but, in contrast, did not predict for relapsed disease. This distinction may reflect underlying biological differences in the pathways toward relapsed versus refractory disease, and further, could have clinical implications. In contrast with refractory disease, relapsed disease occurs later in follow-up, after a disease-free interval. With a longer latency before disease failure, it is possible that early data from the initial PET is less predictive of the relapse outcome. Future studies exploring associations between radiomic biomarkers and relapse may benefit from analyses of interim or post-treatment imaging results. Clinical data also suggest that relapsed and refractory disease may be distinct processes. For example, the long-term survival for patients with relapsed disease tends to be more favorable. It ranged from 27% to 83% in the German Hodgkin Study Group trials, depending on clinical risk factors^36,37^. In contrast, for patients with refractory disease, long-term survival ranges between 8% and 50%^3,4^. Therefore, the imaging biomarker features on initial staging PET scans may provide an early clue as to fundamentally differing biological behaviors of relapsed versus refractory disease and response to current systemic agents.

The optimal method for tumor volume segmentation is debated. We used a fixed threshold for tumor delineation. This method yields reproducible volumes; however, a disadvantage of the fixed threshold approach is that tumor with low FDG-uptake is excluded, which may bias heterogeneity assessments. Additionally, this approach does not take background uptake into consideration^44^. In future work, segmentation methods may be compared to identify the preferred approach in this patient population.

Our hypothesis-generating study is not without limitations. For example, PET technology evolved over the course of the study period, so some scans were obtained in 2D mode and others in 3D mode. Significant care was taken to minimize differences between scans obtained in 2D versus 3D mode; however, we cannot exclude the possibility that PET acquisition mode influenced the radiomic features and, thus, affected our findings.

A second critical limitation is that our findings are based on a single institutional dataset with a small number of events and have not been validated using an external cohort. Radiomic features may be influenced by PET acquisition and reconstruction parameters^45–48^. Therefore, models based on data from one scanner or institution may not be directly applicable to data from another scanner or institution. Methods to standardize PET scanning have been proposed^49,50^; nonetheless, variability across institutions exists. External validation of our model is an important next step. Its robustness and reproducibility must be confirmed, before it can be applied in clinical practice. One PET acquisition factor that warrants particular attention in mediastinal lymphoma texture feature analysis is respiratory motion, which may result in blurring across the tumor volume^51,52^.

Despite these limitations, to the best of our knowledge, this is the first study to demonstrate an association between advanced PET radiomic features and refractory disease status in early-stage HL patients. We suggest that our model be tested in future studies. We used an open-source platform for feature extraction, so researchers at other institutions can explore our findings. Development and validation of an imaging biomarker-based prognostic schema could aid a risk-adapted, individualized therapeutic approach.

## Data Availability

The datasets generated and analyzed during the current study are available from the corresponding author on request.
